# Biological pathway analysis by ArrayUnlock and Ingenuity Pathway Analysis

**DOI:** 10.1186/1753-6561-3-S4-S6

**Published:** 2009-07-16

**Authors:** Ángeles Jiménez-Marín, Melania Collado-Romero, María Ramirez-Boo, Cristina Arce, Juan J Garrido

**Affiliations:** 1 Grupo de Genómica y Mejora Animal, Departamento de Genética, Facultad de Veterinaria, Universidad de Córdoba, Campus de Rabanales, Edificio C-5, 14071 Córdoba, Spain

## Abstract

**Background:**

Once a list of differentially expressed genes has been identified from a microarray experiment, a subsequent post-analysis task is required in order to find the main biological processes associated to the experimental system. This paper describes two pathways analysis tools, ArrayUnlock and Ingenuity Pathways Analysis (IPA) to deal with the post-analyses of microarray data, in the context of the EADGENE and SABRE post-analysis workshop. Dataset employed in this study proceeded from an experimental chicken infection performed to study the host reactions after a homologous or heterologous secondary challenge with two species of *Eimeria*.

**Results:**

Analysis of the same microarray data source employing both commercial pathway analysis tools in parallel let to identify several biological and/or molecular functions altered in the chicken *Eimeria maxima *infection model, including several immune system related pathways. Biological functions differentially altered in the homologous and heterologous second infection were identified. Similarly, the effect of the timing in a homologous second infection was characterized by several biological functions.

**Conclusion:**

Functional analysis with ArrayUnlock and IPA provided information related to functional differences with the three comparisons of the chicken infection leading to similar conclusions. ArrayUnlock let an improvement of the annotations of the chicken genome adding InterPro annotations to the data set file. IPA provides two powerful tools to understand the pathway analysis results: the networks and canonical pathways that showed several pathways related to an adaptative immune response.

## Background

Microarray provides expression levels for thousands of genes simultaneously. The differentially expressed genes can be studied with different pathway analysis tools to connect with existing biological pathways by using public sources. Therefore, the integration of the differentially expressed genes into known biological pathways is a versatile tool for understand the biological complexity of gene expression. The EADGENE and SABRE post-analysis workshop evaluated different methods and software to deal with the post-analysis of microarray data [1]. In this study the analysis tools employed were Array Unlock an IPA and the data set used comes from microarrays assays performed to characterize the gene expression profile after a homologous or heterologous challenge of broilers primed with *Eimeria maxima *as summarised in [1].

## Methods

### Microarray dataset

The microarray employed in this study was the Arkgenomics chicken 20 K oligo microarray prepared from 20,460 oligonucleotides designed against the chicken ENSEMBL transcripts [2].

### Experiment

Two weeks old chicken infected with *Eimeria maxima *were challenged two weeks later with *Eimeria maxima *(MM), *Eimeria acervulina *(MA), or PBS (PM). The samples were collected at 8 hours (MM8, MA8, PM8) and 24 (MM24) hours after infection. The analysis performed allow us to obtain information about: i) differences among a homologous second infection or a heterologous one with another specie of *Eimeria *(MM8_MA8); ii) how changes the response along the time after a second homologous immunization (MM8_MM24); and iii) the secondary immune response (MM8_PM8) [1].

### Working file

The three lists of genes differentially expressed were previously filtered by an adjusted p-value < 0.05. Three working files were generated to perform both analyses using the three datasets. These files must contain one column including all gene ID annotations identified by the two bioinformatics tools. This column was generated according to the annotations provided in the annotation file: original gene IDs (Unigene, HGNC) and mapped with human, mouse and rat homolog. Additional file 1 contains the 'working files' for the three comparisons.

### ArrayUnlock software

ArrayUnlock (Integromics S.L., Spain) [3] was used to explore the main biological processes associated to chicken infection employing the 'Biological Enrichment' functionality. This functionality is able to find those biological annotations that are highly associated to a list of genes differentially expressed. Selected annotations were GO Biological Process, GO Molecular Function, GO Cellular Component KEGG pathways and INTERPRO motifs. Annotation associations were filtered by a p-value ≤ 0.01.

### Ingenuity Pathways Analysis

The ''Core Analysis' function included in IPA (Ingenuity System Inc, USA) [4]  was used to interpret the chicken data in the context of biological processes, pathways and networks. All Identifier Types were selected since more than one type of identifier exists in our dataset (working file). Both up- and down-regulated identifiers were defined as value parameters for the analysis. After the analysis, generated networks are ordered by a score meaning significance. On the other side, significance of the biofunctions and the canonical pathways were tested by the Fisher Exact test p-value. Biofunctions were grouped in: Disease and Disorders; Molecular and Cellular Functions; and, Physiological System Development and Function. In a similar way canonical pathways were grouped in Metabolic Pathways and Signaling Pathways. Canonical pathways can also been ordered by the ratio value (number of molecules in a given pathway that meet cut criteria, divided by total number of molecules that make up that pathway). In contrast to ArrayUnlock, this pathway analysis tool generates networks where the differentially regulated genes can be related according to previously known associations between genes or proteins, but independently of established canonical pathways. Moreover, networks are associated to functions according to the molecules involves.

## Results

### Functional analysis and biological enrichment by ArrayUnlock

Functional analysis results using ArrayUnlock identified significant biological functions altered differentially in the three comparisons analyzed. For each of the biological annotations groups an Excel file was generated including the complete information obtained for each comparison (See Additional files 2, 3, 4, 5, 6). Interestingly, this pathway analysis tool let us 'enrich' gene annotations with Interpro motifs annotations. Results were also visualized as pie and horizontal-bar charts including the 20 most significant associated biological functions (results not shown, see Additional file 7 as an example). A summary of pie and horizontal-bar charts information is presented in Tables 1 and 2 for Biological Processes and KEGG pathways ordered by significance and number of implicated genes. The low number of genes significantly associated to this functions in the comparison MM8_MA8 (most of them among 1 and 4 genes) denotes low differences among a homologous and a heterologous second immunization. However, a higher number of differentially expressed genes where associated to biological functions in comparison MM8_MM24, showing a clear different response to a homologous second immunization associated to the time. On the other hand, the results obtained in MM8_PM8 for KEGG (Table 2) show pathways differentially altered among a primary and a secondary immune response. Most of these pathways were no present in the other two comparisons which shows that in both MA8 and MM24 such a typical secondary response was developed as in MM8.

**Table 1:** Top ten Biological Processes significantly altered in ArrayUnlock analysis. In brackets, number of genes from the input file implicated in each annotation. Significance at p < 0.05.

Biological Process		
MM8_MA8	MM8_MM24	MM8_PM8

- Regulation of transcription DNA-dependent (5)	- Signal transduction (34)	- Signal transduction (84)
- Metabolic process (4)	- Transcription (24)	- Regulation of transcription, DNA-dependent (71)
- Cell adhesion (4)	- Cell adhesion (19)	-Transcription (65)
- Transcription (4)	-Multicellular organismal development (18)	- Multicellular organismal development (44)
- Actin cytoskeleton organization and biogenesis (4)	- Ion transport (15)	- Cell cycle (34)
- Cytoskeleton organization and biogenesis (2)	- Protein amino acid phosphorylation (13)	- Protein transport (33)
- Chromatin modification (2)	- Cell differentiation (12)	- Metabolic process (30)
- Small GTPase mediated signal transduction (2)	- Nervous system development (12)	- Apoptosis (29)
-Integrin-mediated signalling pathway (2)	- Cell cycle (12)	- Cell adhesion (28)
-Cation transport (2)	- Protein transport (11)	- Protein amino acid phosphorylation (28)

**Table 2:** Top ten KEGG Pathways significantly altered in ArrayUnlock analysis. In brackets, number of genes from the input file implicated in each annotation. Significance at p < 0.05.

KEGG Pathways		
MM8_MA8	MM8_MM24	MM8_PM8

- GnRH signalling (1)	- MAPK signalling pathway (13)	- Focal adhesion (22)
- Regulation of actin cytoskeleton (1)	-Neuroactive ligand-receptor interaction (10)	- MAPK signalling pathway (21)
- Long-term potentiation (1)	- Regulation of actin cytoskeleton (8)	-Jak-STAT signalling pathway (16)
- Leukocyte transendothelial migration (1)	- Focal adhesion (8)	- Cytokine-cytokine receptor interaction (16)
- Focal adhesion (1)	- GnRH signalling pathway (6)	- Cell cycle (14)
- Ubiquitin mediated proteolysis (1)	- Axon guidance (6)	- Fc epsilon RI signalling pathway (11)
- MAPK signalling pathway(1)	- Calcium signalling pathway (6)	- Natural killer cell mediated cytotoxicity (11)
- Glycan structures-degradation (1)	- Pancreatic cancer (4)	- Insulin signalling pathway (10)
- Glycan structures-biosynthesis 2 (1)	- Long-term potentiation (4)	- Apoptosis (10)
- Glycan structures-biosynthesis 1 (1)	-Leukocyte transendothelial migration (4)	- T cell receptor signalling pathway (9)

### Functional analysis by IPA

**Figure 1:**
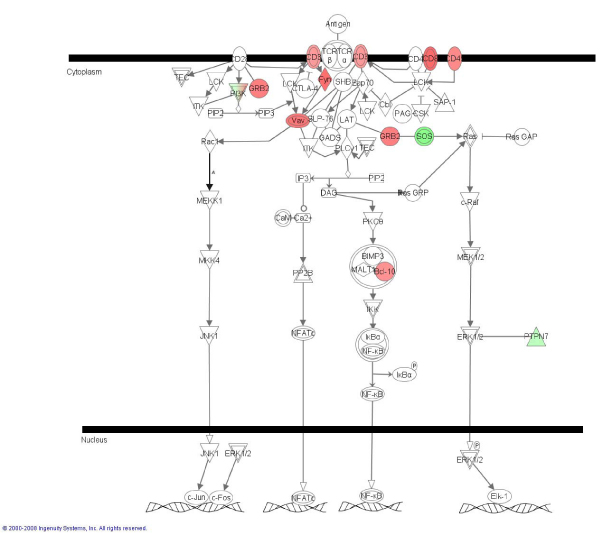
**T cell receptor signalling canonical pathway obtained by IPA obtained in comparison MM8_PM8**. Up- and down-regulated genes in red and green, respectively.

IPA identified significant networks, top functions and canonical pathways associated with the differentially expressed genes for each comparison analyzed (see Additional file 8). The first networks scored for the MM8_MM24 and MM8_PM8 comparisons are presented in Additional file 9. A similar result to that obtained with ArrayUnlock was obtained for the comparison MM8_MA8, a lower number of genes significantly associated to biological functions (a maximum of five genes per function) compared with the other two comparisons. Similarly, in this comparison, only five canonical pathways were significant. In the comparison MM8_MM24 seven out of the ten most significant canonical pathways were related to cellular signalling, e.g.: cAMP signalling; integrin signalling; actin cytoskeleton mediated signalling; and G-coupled receptor signaling. Interestingly, in this comparison the functions more significant and with higher number of genes implicated correspond to 'cell morphology', 'cellular assembly and organization', and 'cellular development' being most genes down-regulated. In the comparison MM8_PM8 the 'immune response' and 'immune and lymphatic system development and function' are among the most significant functions altered. Then, most genes related to proliferation and maturation of B lymphocytes, recruitment of macrophages and antigen presenting cells, increasing of NK cells and T-cells were up-regulated. As an example, the T cell receptor signalling canonical pathway obtained by IPA and associated with the differentially expressed genes for the comparison MM8_PM8 is shown Figure 1.

## Conclusion

The results of the analysis were highly dependent of having the most complete annotation available in the data set file. According to this, the creation of the 'working file' was critical in order to take the maximum advantage of the analysis what can be considered as a drawback of both tools.

Both tools provided, information for a global understanding of the underlying biological processes, independently. First, homologous and heterologous second infection induces similar changes in gene expression, although some differences were found associated to several biological functions. Second, the response upon a second homologous infection varied with the time and differed significantly in a relative high number of biological functions. And third, a core of biological functions and pathways associated to a secondary response were similar when the second challenge varied in the time and also in the case of a heterologous secondary infection.

The two analytical tools provided overlapping information so as complementary information. Main differences were due to databases used for each tool. UrrayUnlock results are based in gene ontology terms or KEGG annotations widely known and used in other analytical tools and able to be consulted in free-access databases. On the other side, IPA makes use of a non public bibliographic database and own terminology for functions classification that not always are directly correlated with GO terms. An advantage of Ingenuity was that this tool classify the genes implicated in each function within sub-functions and provide direct link of each molecule to the bibliographic reference were that relationship is described. The results obtained for both tool to identify altered established pathways (canonical pathways in IPA and KEGG pathways in ArrayUnlock) were similar, however, IPA integrates the information of the differentially expressed genes within the figures highlighting the up or down regulation. In general, IPA provided a better presentation of the results and an easier identification of molecules implicated in each function within the interface of the software. Moreover, IPA generates networks where the differentially regulated genes can be related according to previously known associations between genes or proteins, but independently of established canonical pathways.

## Competing interests

The authors declare that they have no competing interests.

## Authors' contributions

MRB learned the management of the two tools and trained to the rest member's group. The comparisons were analyzed by MRB, CA, MCR and AJM. AJM conducted the documentation and communication related to the workshop to the other group members. JJG coordinated and supervised the working team.

## Supplementary Material

Additional file 1 **Working files**. Gene annotation, fold-change, and the identifier of the original data are shown. Click here for file

Additional file 2 **ArrayUnlock Biological Process**. File contains information about: biological function annotation; p-value and corrected p-value; number of genes of our list implicated in each annotation; number of gene from the ArrayUnlock database implicated in this annotation; and finally, a description of each GO biological function annotation. Click here for file

Additional file 3 **ArrayUnlock Molecular Functions**. File contains information about: biological function annotation; p-Value and corrected p-Value; number of genes of our list implicated in each annotation; number of gene from the ArrayUnlock database implicated in this annotation; and finally, a description of each GO biological function annotation. Click here for file

Additional file 4 **ArrayUnlock Cellular Components**. File contains information about: biological function annotation; p-Value and corrected p-Value; number of genes of our list implicated in each annotation; number of gene from the ArrayUnlock database implicated in this annotation; and finally, a description of each GO biological function annotation. Click here for file

Additional file 5 **ArrayUnlock KEGG Pathways**. File contains information about: biological function annotation; p-Value and corrected p-Value; number of genes of our list implicated in each annotation; number of gene from the ArrayUnlock database implicated in this annotation; and finally, a description of each GO biological function annotation. Click here for file

Additional file 6 **ArrayUnlock INTERPROMotifs**. File contains information about: biological function annotation; p-Value and corrected p-Value; number of genes of our list implicated in each annotation; number of gene from the ArrayUnlock database implicated in this annotation; and finally, a description of each GO biological function annotation. Click here for file

Additional file 7 Graphical representation as horizontal-bar (A) and pie chart (B) of the results obtained for Biological Process using ArrayUnlock software for the comparison MM8_PM8. Click here for file

Additional file 8 IPA top Networks, BioFunctions and Canonical Pathways for the three comparisons analyzed. Click here for file

Additional file 9 The first network identified by IPA analysis for MM8_MM24 and MM8-PM8 analysis. Click here for file
