# Supplementary material for: Biological pathway analysis by ArrayUnlock and Ingenuity Pathway Analysis
Source: BMC Proc. 2009 Jul 16;3(Suppl 4):S6. doi: 10.1186/1753-6561-3-S4-S6 (PMC2712749; doi:10.1186/1753-6561-3-S4-S6)

Additional file 7. Graphical representation as horizontal-bar (A) and pie chart (B) of the results obtained for Biological Process using ArrayUnlock software for the comparison MM8_PM8.

A


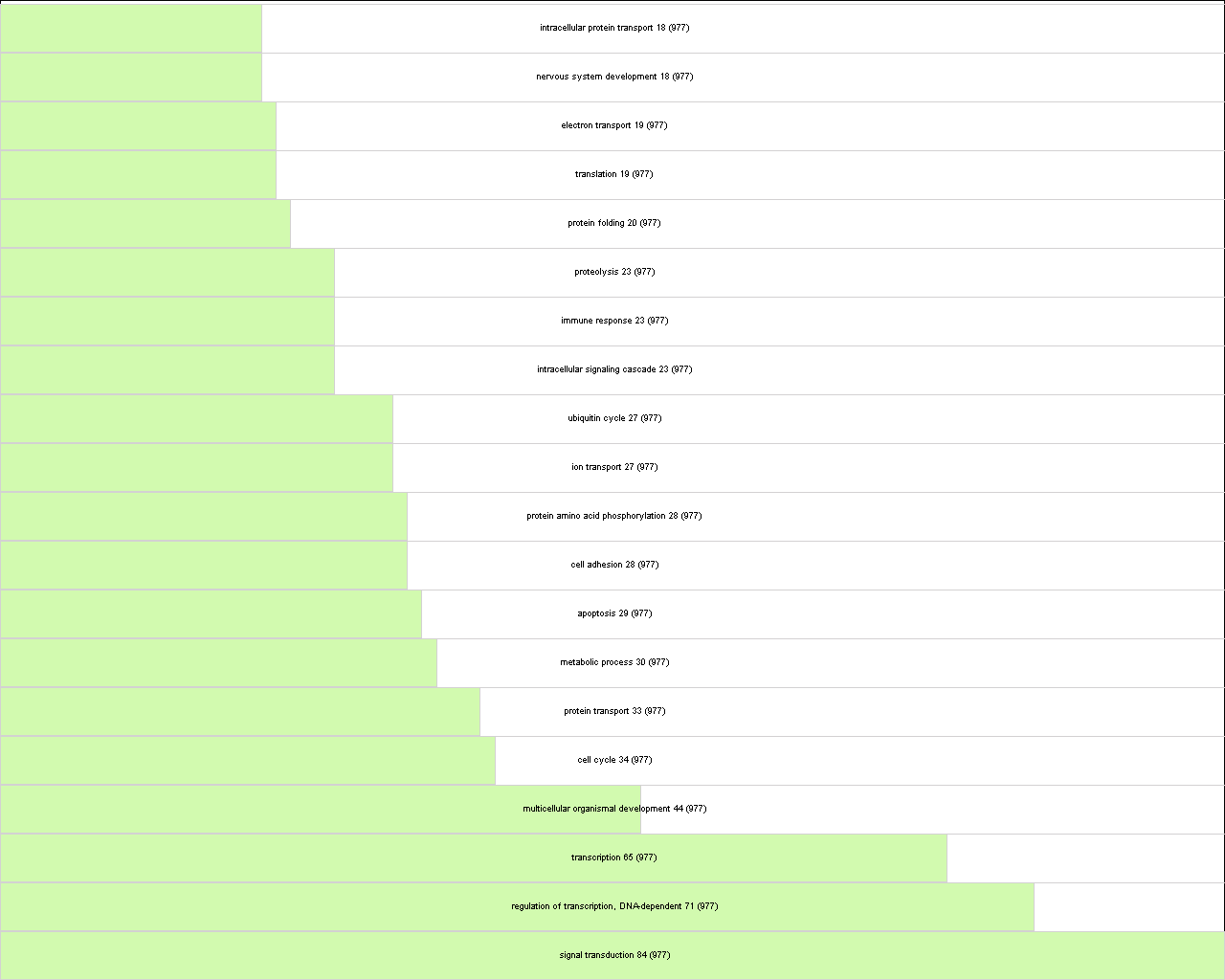


B


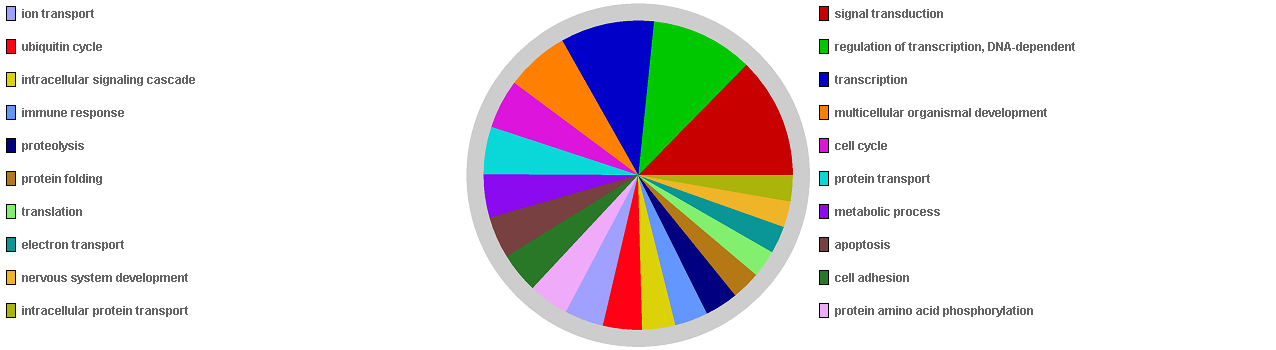

Supplement: Additional file 7 — Graphical representation as horizontal-bar (A) and pie chart (B) of the results obtained for Biological Process using ArrayUnlock software for the comparison MM8_PM8. [file 1753-6561-3-S4-S6-S7.doc]
