# Supplementary material for: Biological pathway analysis by ArrayUnlock and Ingenuity Pathway Analysis
Source: BMC Proc. 2009 Jul 16;3(Suppl 4):S6. doi: 10.1186/1753-6561-3-S4-S6 (PMC2712749; doi:10.1186/1753-6561-3-S4-S6)

**Additional file 9.**

**The first network identified by IPA analysis for MM8_PM8 comparison and related to the bio functions Cancer, Gastrointestinal Disease and Tumour Morphology.** In green, down-regulated genes; in red, up-regulated genes.


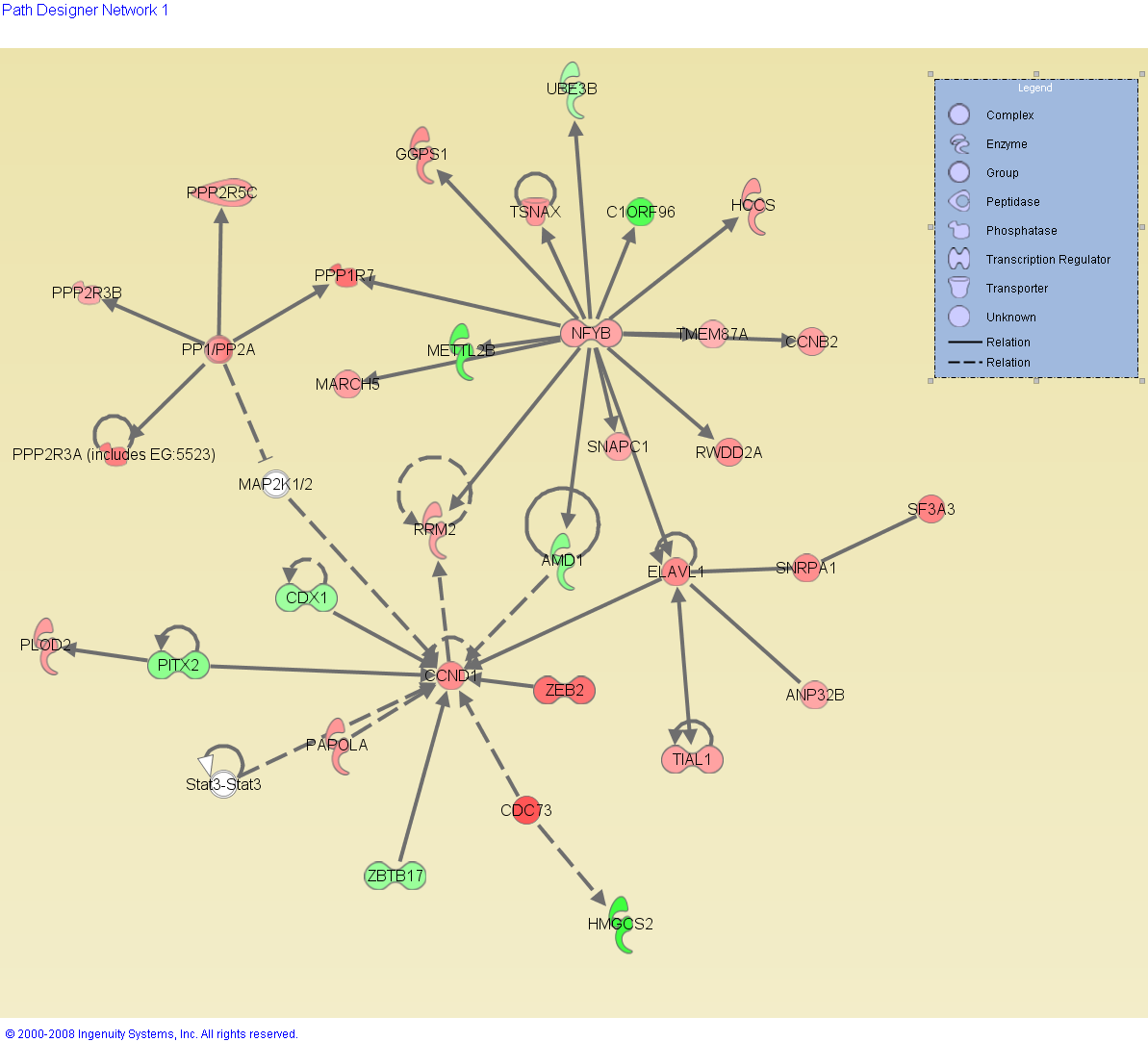


**The first network identified by IPA analysis for MM8_MM24 comparison and related to the bio functions Cell death, Immunological Disease and Connective tissues disorders.** In green, down-regulated genes; in red, up-regulated genes.


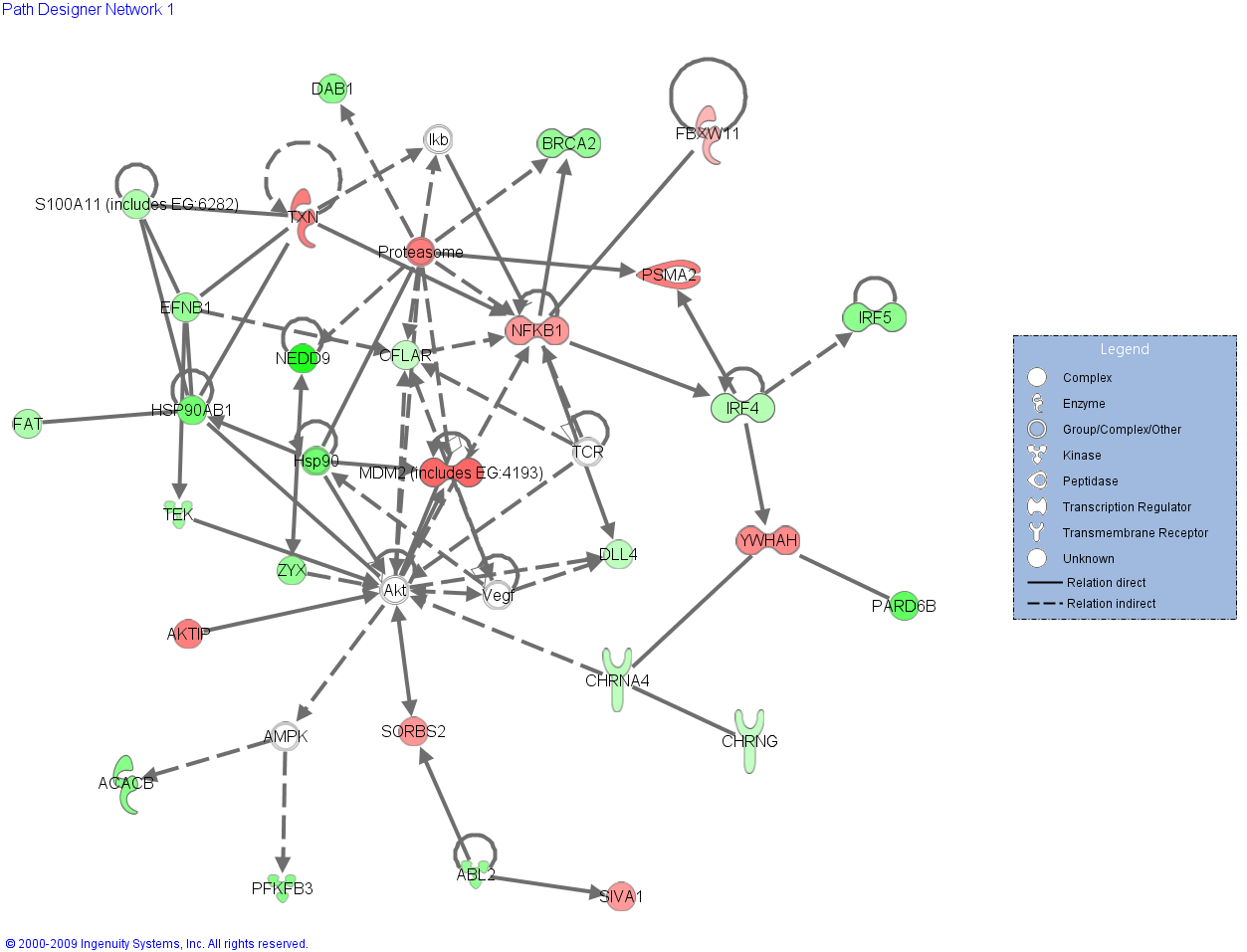

Supplement: Additional file 9 — The first network identified by IPA analysis for MM8_MM24 and MM8-PM8 analysis. [file 1753-6561-3-S4-S6-S9.doc]
